# Using the RE-AIM Framework to Evaluate a Physical Activity Intervention in Churches

**Published:** 2007-09-15

**Authors:** Melissa Bopp, Sara Wilcox, Stephen P Hooker, Kimberly Butler, Lottie McClorin, Marilyn Laken, Ruth Saunders, Deborah Parra-Medina

**Affiliations:** Kansas State University; Department of Exercise Science, Arnold School of Public Health, University of South Carolina, Columbia, South Carolina; Department of Exercise Science, Arnold School of Public Health, University of South Carolina, Columbia, South Carolina; Department of Exercise Science, Arnold School of Public Health, University of South Carolina, Columbia, South Carolina; Department of Exercise Science, Arnold School of Public Health, University of South Carolina, Columbia, South Carolina; Office of Special Initiatives, College of Nursing, Medical University of South Carolina, Charleston, South Carolina; Department of Health Promotion, Education and Behavior, Arnold School of Public Health, University of South Carolina, Columbia, South Carolina; Department of Health Promotion, Education and Behavior, Arnold School of Public Health, University of South Carolina, Columbia, South Carolina

## Abstract

**Introduction:**

Health-e-AME was a 3-year intervention designed to promote physical activity at African Methodist Episcopal churches across South Carolina. It is based on a community-participation model designed to disseminate interventions through trained volunteer health directors.

**Methods:**

We used the RE-AIM (Reach, Effectiveness, Adoption, Implementation, and Maintenance) framework to evaluate this intervention through interviews with 50 health directors.

**Results:**

Eighty percent of the churches that had a health director trained during the first year of the intervention and 52% of churches that had a health director trained during the second year adopted at least one component of the intervention. Lack of motivation or commitment from the congregation was the most common barrier to adoption. Intervention activities reached middle-aged women mainly. The intervention was moderately well implemented, and adherence to its principles was adequate. Maintenance analyses showed that individual participants in the intervention's physical activity components continued their participation as long as the church offered them, but churches had difficulties continuing to offer physical activity sessions. The effectiveness analysis showed that the intervention produced promising, but not significant, trends in levels of physical activity.

**Conclusion:**

Our use of the RE-AIM framework to evaluate this intervention serves as a model for a comprehensive evaluation of the health effects of community programs to promote health.

## Introduction

Participating in regular physical activity (PA) results in many physical and mental health benefits (1), including reduced risk for chronic diseases such as cardiovascular disease, diabetes mellitus, obesity, and certain types of cancer. PA also has mental health benefits, including decreased symptoms of depression and anxiety. Despite the benefits of engaging in regular PA, rates of doing so are low, especially among certain groups (e.g., women, the elderly, and people from minority racial or ethnic populations) (2).

Although interventions to increase PA are effective, relatively few include African Americans as participants, and fewer still are designed specifically for African Americans. A recent review by Yancey and colleagues (3) of community strategies for increasing PA by ethnically diverse populations found that, before 1990, PA intervention research seldom included African Americans. However, the focus has shifted in recent years to include more communities of color. The researchers (3) describe the important elements of community research: they include coalition building, mass media campaigns, and community partnerships. They also found that providing information and resources to promote health and foster coalitions and networks were the approaches most commonly used in community PA interventions that target ethnically diverse groups (3). The Yancey review found some interventions that were successful in promoting PA among African Americans, but the information available on how best to increase PA by African Americans is limited, and there is no consensus on the best approach, theory, or framework to use.

Health promotion programs in churches have enjoyed moderate success among African Americans (4-10). The focus of these programs is improving dietary habits, increasing PA, reducing smoking, managing chronic disease, screening for behaviors that increase risk for chronic disease, and managing risk factors that already exist. In a review of 28 interventions affiliated with churches, 41.5% targeted the health-related behaviors of African Americans, including behaviors that 1) affect general health, 2) increase risk for cardiovascular disease, and 3) increase risk for cancer (11). The churches were involved in the interventions to varying degrees, ranging from simply hosting the program on their premises to full-scale partnering with the intervention team to develop, implement, and evaluate the intervention activities (11).

Interventions to promote PA are often designed for tightly controlled research settings with generous resources and materials to allow for large effects. Translating these interventions to practice allows for a greater public health effect, although the feasibility of producing the same results is often unknown (12). The recent focus on translating research projects into public health interventions (13) led to the development of the RE-AIM (Reach, Effectiveness, Adoption, Implementation, Maintenance) framework to assess the effects of public health programs that were developed, implemented, and evaluated in a tightly controlled environment and then disseminated for wider use in communities (14). *Reach* is the proportion of eligible people in the target population who participate in an intervention and the extent to which those participants represent the target population. *Effectiveness* is the extent to which the intervention has a positive effect on relevant outcomes. *Adoption* is an organizational measure of the number of program providers who implement a program and the extent to which they represent all possible program providers. *Implementation* is an organizational measure of the quality of the intervention's delivery and its adherence to the essential elements of the research program. *Implementation* is sometimes called *treatment fidelity*. *Maintenance* is a measure of the intervention's effectiveness at achieving the desired outcome for an extended time. It is also a measure of the sustainability of a program and indicates whether a program is likely to become institutionalized (14).

Most evaluations do not assess all RE-AIM components: most assess *reach* and *effectiveness*, and some assess *adoption, implementation,* or* maintenance* at individual or organizational levels (14,15). Success at translating behavioral programs into public health practice means that we must pay close attention to the elements of a program that can most easily be translated into practice (12). With that objective in mind, we assessed the public health effects of the Health-e-AME Physical-e-Fit intervention using the RE-AIM framework of program evaluation.

### The Health-e-AME Physical-e-Fit program

The Health-e-AME Physical-e-Fit program was a 3-year community PA intervention, which is described in detail elsewhere (16). The intervention was developed in partnership with South Carolina's African Methodist Episcopal (AME) Church Planning Committee so that it would reflect the needs and interests of members. The intervention had a delayed-participation design, which meant that about half of the state's AME churches were eligible to participate in the intervention during the first year and half were eligible during the second year. All churches were eligible to participate during the third year. The main goals of the Physical-e-Fit program were to increase awareness of the importance of PA, increase church members' participation in PA, and have key leaders within the church emphasize and promote PA.

Several intervention activities were developed: praise aerobics (i.e., aerobics set to gospel music), chair exercises, walking programs, and a behavior and skill-based class that focused on helping participants become active and eat healthfully. The Physical-e-Fit program also had educational messages about PA posted within the church and built PA into other church activities. All activities and messages had spiritual or religious components involving scripture and culturally specific materials.

### Training health directors and physical activity coordinators

The Health-e-AME project staff trained health directors (HDs) or physical activity coordinators (PACs) to organize and deliver the intervention within their churches' health ministry. HDs are generally responsible for all health-related activities in their churches, and PACs are responsible only for the Physical-e-Fit program. Usually PACs are in churches that do not have an established health ministry or in churches that have a health ministry large enough to delegate responsibilities to different people. Churches select which intervention activities to adopt on the basis of the resources available and the interests of their congregation.

The training sessions that the HDs and PACs attended were held at churches and lasted 2 to 4 hours. Attendees were given didactic and hands-on learning experiences with PA demonstrations and participation. The sessions covered the basics of PA and healthy eating, details of the physical activity components of the intervention, ideas for educational activities, and tips for getting their pastors and congregations interested and motivated. The HDs and PACs were given simple manuals on how to implement the intervention at their churches and incentives to share with the congregation. Technical assistance was also provided after the trainings.

## Methods

We selected for interview a random sample of 50 trained HDs or PACs (25 trained during the first year of the intervention and 25 trained during the second year) from all trained HDs and PACs. The interviewees consented verbally to participate. Trained project staff conducted the interviews and took detailed interview notes. Also collected was information about the church, including size (small, <50 congregants; medium, 50-100; large, >100) and congregation composition (age groups, sex ratios).

An interview guide was developed so that we could collect data and assess each component of RE-AIM. The guide consisted of open-ended questions asking HDs and PACs to describe details about their program activities (if any), barriers they encountered, and their successes and struggles with offering the Physical-e-Fit program. Additional details about the interview guide are given in the next section with the description of how we assessed each component of the RE-AIM framework.

### Reach

To assess *reach* (the proportion of eligible people in a church's congregation who participated in the Health-e-AME Physical-e-Fit intervention), we asked interviewees to estimate the number of participants in their Health-e-AME Physical-e-Fit programs and to describe the demographic characteristics (i.e., sex and age group) of the participants. We analyzed separately the data on women, people aged 41 to 64, and people aged 65 or older. The number of churches with trained HDs or PACs was also recorded.

### Effectiveness

Given the large size of the Health-e-AME Physical-e-Fit program, detailed information on the effectiveness analyses of this intervention is beyond the scope of this article. However, measuring its effects on levels of PA is described in detail elsewhere (17). In brief, the intervention was evaluated through a telephone survey of randomly selected church members (n = 571) from 20 AME churches. At baseline, year 1, and year 2, the Behavioral Risk Factor Surveillance System's PA module (18) was used to assess moderate PA participation by church members and the percentage of church members who engaged in PA at recommended levels.

### Adoption

We estimated the adoption rate (i.e., the proportion of churches with trained HDs or PACs that began the Health-e-AME Physical-e-Fit program) through interviews with the HDs and PACs. Interviewers assessed whether the interviewees implemented the program correctly. If the interviewers determined that the program was not implemented correctly, they asked what barriers the HDs and PACs met and how they planned to overcome those barriers. We also estimated the adoption rate by church size, geographic location, and characteristics of the church neighborhood as determined by census data (19).

### Implementation

We measured the level of implementation (i.e., fidelity to delivering the Health-e-AME Physical-e-Fit program as the developers intended) at churches that had at least one PA program. The interviewers went through a checklist of principles for the Physical-e-Fit programs with the interviewees and determined how well each church adhered to the program's principles when implementing the intervention.

### Maintenance

We assessed both organizational and individual maintenance. That is, we determined the extent to which the Health-e-AME Physical-e-Fit program became integrated into the activities of the churches that had a trained HD or PAC, and we determined the extent to which individuals who enrolled in intervention activities continued with those activities. To do so, we interviewed only the 25 trained HDs and PACs from year 1 of the intervention. The interviewers assessed whether the interviewees had taken one of three actions: 1) implemented intervention activities that were still ongoing, 2) implemented intervention activities that were no longer being offered at the church, or 3) had never implemented any intervention activities. Interviewees who were still offering intervention activities described 1) their challenges to continuing to offer the activities and 2) their plans for continuing to offer them in the future. Those who had once offered intervention activities but stopped described what prevented them from continuing to offer the program. On the basis of data collected through these interviews, we assessed the percentage of churches who continued to participate in the program.

We assessed individual maintenance in the program by asking the HDs and PACs to estimate the length of participation in PA by individuals involved in any PA activity. On the basis of these estimates, we assessed the percentage of continued individual participation in the programs.

## Results

We contacted 76 HDs or PACs with a request for an interview and actually interviewed 50. All interviewees were women, which was representative of all of trainees, whom we estimated to be 95% female. We were unable to interview 26 HDs or PACs for the following reasons: unable to reach after repeated attempts (n = 14), telephone number incorrect or disconnected (n = 6), no longer attending the church (n = 2), unwilling to participate (n = 2), and reason unknown (n = 2). Table 1 describes the characteristics of the churches with HDs or PACs who were interviewed. The mean number of HDs or PACs trained per church was two, and the mean household income for the neighborhoods of all churches surveyed was $32,473. The interviews were done during the third year of the intervention, from November 2004 through January 2005.

### Reach

A total of 889 congregants from 303 churches, about 50% of all AME churches in South Carolina, were trained in the activities and principles of the intervention. Because reach data were skewed, we report only ranges and medians. Overall reach (the proportion of church members who participated in PA sessions) among the churches that participated in the program ranged from 2% to 100% with a median of 18.5%.

Reach was also calculated to assess representativeness. On the basis of 50 interviews, reach among adult women ranged from 2% to 100% (median, 20%); reach among adults aged 65 or older ranged from 0% to 100% (median, 8%); and reach among adults aged 41 through 64 ranged from 0% to 100% (median, 11%). Data on men were not analyzed because interviewees reported minimal participation by men in any activity. Interviewees reported that their congregations were on average 58% female; 31% were 41 to 64, and 39% were 65 or older.

### Effectiveness

As previously reported, 418 (73%) people from the baseline cohort of randomly selected church members completed the 1-year and 316 (55%) completed the 2-year follow-up telephone surveys (18). The intervention had no significant effect on getting people to follow public health recommendations for PA (*P* = .08). Interviewees who had heard of Health-e-AME were significantly more likely to report engaging in some type of PA at the 1- and 2-year follow-ups, and were significantly more likely to be following PA recommendations at the 2-year follow-up interview than at the 1-year interview.

### Adoption

Rates of adoption differed by year of adoption: 80% of churches with HDs or PAC trained in year 1 adopted the program, but only 52% of churches with HDs or PACs trained in year 2 adopted the program. Overall, 6 of 13 small, 8 of 15 medium, and 19 of 22 large churches adopted the program; 7 of 13 small, 7 of 15 medium, and 3 of 22 large churches did not adopt. Large churches were more likely than medium or small churches to offer two or more types of physical activity: 8 of 18 large churches, 2 of 6 medium churches, and 2 of 6 small churches did so. We found no difference in adoption rates on the basis of geographic area (urban or rural), congregants' ethnicity or race, or the socioeconomic status of the census tract in which the church was located. Reported barriers to adoption at churches with more than one person trained in the Health-e-AME intervention were not different from the barriers faced by churches with only one person trained.

The most commonly reported challenges at churches that adopted the program were lack of motivation or commitment from the congregation (45%), problems related to the pastor (e.g., turnover, lack of support for the program) (24%), and problems related to the health director (e.g., health problems, family or work commitments) (18%). Among churches that did not adopt the program, issues related to the HDs or PACs (e.g., health problems, family or work commitments) were the most common barriers (80%). Also stated as barriers were church-related factors (e.g., problems implementing the program because of other competing church events or the physical design or layout of the church) (60%) and lack of motivation and commitment from the congregation (60%).

### Implementation

Implementation results are shown in Table 2. Walking programs were the most commonly offered, and the behavior-change class (*8 Steps to Fitness*) was the least commonly offered. Adherence to program principles ranged from 50% to 100%. Screening potential participants to ensure they could safely engage in praise aerobics was the principle least adhered to.

### Maintenance

HDs and PACs trained during year 1 estimated the length that individuals participated in the PA components of the intervention. HDs and PACs from the 13 churches (52%) that maintained their programs throughout year 1 indicated that most participants who enrolled when the program began stayed with the program.

Of the 25 churches with HDs or PACs who were trained during the first year, 13 were still offering at least one PA component when we interviewed the HDs and PACs, 7 had begun the intervention but were no longer offering any components, and 5 had never offered any components. The 13 churches that were still offering intervention components most often cited lack of motivation or commitment from the congregation (38%) as their greatest challenge to maintaining it. The primary reasons (71%) that seven churches stopped offering PA components were problems related to the HDs or PACs (e.g., health problems, family or work commitments).

## Discussion

This study was done in response to 1) the recent call for evaluations of how research findings translate into large-scale projects and 2) the need for understanding the logistics of implementing such projects (12,20-22). Our study results provide a comprehensive overview of the implementation and evaluation of the Health-e-AME Physical-e-Fit program. By using the RE-AIM framework, project staff collected valuable formative and summative information that helped with implementing the intervention and providing HDs and PACs with technical assistance. Overall, the intervention was only somewhat effective at increasing PA, and elements of the RE-AIM analysis show why it was not more successful.

We examined the public health effect of the Health-e-AME Physical-e-Fit program using the RE-AIM framework to assess the individual and organizational factors associated with a large public health project with community participation. Although we did formative research with both men and women, the intervention as designed did not reach male AME members, which means that the intervention needs to be adapted to make it more appealing to men. Other public health programs set up in churches found that recruiting the pastor as a role model and supporter of the program can increase participation (23), and this approach may increase interest among the men in the AME churches. Additional approaches could include recruiting more men as HDs and PACs, enlisting the help of men who are leaders in the church, and offering more competitive activities such as basketball. A factor that may have limited our finding on male participation is that all of the interviewed HDs and PACs were women, and they may have been successful at mobilizing their own social network within the church (other women in their age groups), and they may have struggled with how to reach men and how to reach women in age groups other than their own. Reach among all AME churches in the state was good (303 churches, about half of all eligible churches). The effectiveness analysis also related to reach, in that we found that people who had heard of the intervention activities at their church were more likely than those who had not heard of them to engage in some type of physical activity either at the church or elsewhere.

Assessing barriers to adoption while the project was ongoing allowed program planners to modify the training and overcome the most common barriers. During the final year of the program, planners worked to overcome obstacles to getting the program started by adding training on issues such as how to deal with pastor-related problems and how to increase motivation and interest among congregants. In addition, more than one person from a church was trained so that program implementation was not dependent on one person. Our results emphasize that the program's success depended on volunteers, and future intervention planners should consider other approaches to implementing the program in churches (e.g., providing incentives for leading the program, changing the program to ease the burden on the HDs and PACs, or having a paid church member assist with the program). Theories on the capacity of a community to implement and maintain health promotion programs emphasize the need to train and motivate volunteers in order to be successful (24).

To ensure the institutionalization and sustainability of the intervention, it is essential to ensure that volunteers receive adequate training, support, and recognition. Lack of training and recognition may explain some of the challenges churches experience with maintaining health promotion programs over time. Future large-scale interventions could include additional trainings for volunteers on how to overcome barriers, recognize successes, network with other volunteers, and re-energize programs to promote their continuation. Given the size and scope of this project (303 churches statewide), program organizers could not provide the additional training. Organizers of similar interventions with lay health volunteers found that incentives and a large team of church members running the program were useful ways to improve success rates (23,25). The higher rates of adoption by churches enrolled during the first year than by those enrolled during the second year could simply be because the first-year churches had more time than the second-year churches to get their program started rather than because of a fundamental difference between the two sets of churches. The rates of adoption could also have affected the effectiveness analysis, since individuals who had heard of Health-e-AME at their church were more likely than those who had not heard of it to engage in some type of physical activity, at or outside church.

Given the less-than-ideal rates of organizational maintenance, we believe that additional strategies to encourage and support continuation of the intervention are needed. Some ideas for doing so are holding special training sessions for church HDs and PACs every year to deal with common barriers and developing new program activities to increase interest and excitement for the program. Other interventions in churches also struggled with maintenance and developed approaches for ensuring that the interventions continued (9,25,26). Our intervention was large and relied on volunteers to lead the activities and to deal with barriers (e.g., pastor turnover, problems within the congregation), which caused difficulties for continued success. Since Health-e-AME is a community intervention, HDs and PACs must be able to adapt it to fit within a particular church community and prevent it from competing with other church events. Such competition  could result in the less-than-ideal rates of maintenance.

Interventions that produce great environmental and social changes (e.g., create or enhance access to PA facilities) might have a better chance of succeeding than would interventions that promote only PA. This intervention included activities designed to create social and environmental changes, but they were not as successful as we had planned. Perhaps more training on ways to make environmental changes would improve maintenance of the intervention at churches. Individual participants who joined a church's PA program as soon as it was offered maintained their participation; similarly, people who join other church activities (e.g., choir, missionary groups) usually continue participating. Unfortunately, because of the large size and geographical dispersion of this intervention's participants, we were unable to assess maintenance directly through interviews with participants, a stronger method of doing so than the one we used. Future adjustments to the program should include providing activities and events of interest to more members of the church, particularly men and adults older than 65.

This study assessed factors that affect the many layers of this intervention in order to understand individual and organizational participation. The RE-AIM model allowed us to examine comprehensively the intricacies of program design, implementation, and evaluation. A recent review by Klesges et al (27) indicated the need for designing health promotion programs with dissemination in mind. We suggest that considering RE-AIM during planning stages of a new program will result in a more complete program, one that addresses issues associated with improving the external and internal validity of  translating programs from research projects into practical public health interventions. Recent research (12,20,28) cites the need to focus on the public health effect of health promoting programs when considering issues of design and dissemination. This study contributes to the limited body of knowledge on interventions that are translatable and useful beyond a tightly controlled research setting.

This study has some limitations. Although interviewees were selected at random, the cohort may have been biased. HDs and PACs whose programs are successful may be more likely to agree to an interview than those who struggle with their programs. Some HDs and PACs may be reluctant to disclose that they were not successful with their programs for fear of repercussion from their pastor or other church leader. Related to this limitation, because this study relied on self-reported information, the possibility that interviewees gave socially desirable responses must be considered. Interviewees may have wanted to make themselves or their church look successful. Lastly, more than 250 churches had HDs or PACs trained in the Health-e-AME Physical-e-Fit program when the interviews occurred. Therefore, this group of 50 interviewees represented a relatively small sample of trained HDs and PACs. Despite these limitations, these results contribute to a growing body of knowledge about the design, implementation, and evaluation of large interventions implemented in churches to increase levels of PA.

Limited information is available about the RE-AIM framework for health promotion programs conducted in partnership with community organizations. This study provides the groundwork for future community health promotion programs as a model for intervention design, implementation, and dissemination. In addition, the results of this study contributed to the intervention being modified to address barriers to implementation and helped us to understand some reasons for the suboptimal effectiveness of the program.

## Figures and Tables

**Table 1:** Characteristics of a Sample of Churches (N = 50) That Participated in the Health-e-AME Program to Promote Physical Activity, South Carolina, 2003–2005

Characteristic	No. (%)
**Size**	
Small (<50 congregants)	13 (26)
Medium (50–100 congregants)	15 (30)
Large (>100 congregants)	22 (44)
**Neighborhood, by race or ethnicity^a^**	
Mostly African Americans	24 (48)
Mostly whites, Hispanics, or people from racial groups other than African American	26 (52)
**Location**	
Urban	36 (72)
Rural	14 (28)

a U.S. Census Bureau (20).

**Table 2:** Implementation of Intervention Activities at a Sample of Churches (N = 50) That Participated in the Health-e-AME Program to Promote Physical Activity, South Carolina, 2003–2005.

Activity	No. of Churches Offering Activity	Mean Adherence to Program Principles (%)
8 Steps to Fitness	7	90
Walking program	16	88
Praise aerobics	11	61
Chair aerobics	9	88
Educational activities	13	Not assessed
